# Declining age-adjusted surgical incidence of intracranial meningiomas: a 19-year retrospective analysis from an academic hospital

**DOI:** 10.1007/s11060-026-05713-1

**Published:** 2026-07-21

**Authors:** Jenni Määttä, Joonas Laajava, Mika Niemelä, Miikka Korja

**Affiliations:** Department of Neurosurgery, University of Helsinki and Helsinki University Hospital, P.O. Box 266, Helsinki, FI-00029 Finland

**Keywords:** Meningioma, Retrospective cohort, Surgical incidence

## Abstract

**Purpose:**

Intracranial meningiomas are the most common primary intracranial tumors, with rising detection rates due to increased neuroimaging. We examined surgical management trends to determine whether surgical incidence is independent of detection rate and to characterize shifts in surgical indications and patient demographics.

**Methods:**

This retrospective cohort study included 2,278 consecutive adults undergoing primary surgical resection for meningiomas at Helsinki University Hospital (2005–2023). Trends in absolute surgical caseload and patient demographics were analyzed. Surgical incidences were standardized to the European Standard Population (ESP 2013), and age-specific Observed-to-Expected (O/E) ratios were calculated against baseline incidences (2005–2008).

**Results:**

Absolute annual surgical caseload remained stable (range: 83–160 cases; crude incidence 3.8–7.7 per 100,000 person-years; *p* = 0.868), while the median age of operated patients increased significantly by 2.9 months per year (*p* = 0.018). The most common presenting symptoms were seizures (19%) and visual symptoms (14%); symptom relief was the most common surgical indication (62%). Age-standardized (ESP) surgical incidences declined over time (3-year sliding average *p* < 0.006 for all age groups), and demographic-adjusted O/E ratio analysis supports this decline, particularly in the 30–69 years age range. Prophylactic surgeries declined significantly (*p* < 0.001), while surgeries prompted by tumor growth (*p* = 0.031) and large tumor size (*p* < 0.001) increased.

**Conclusion:**

Despite increased detection and an aging population, age-adjusted surgical incidence for meningiomas declined over 19 years, supporting the interpretation of a shift toward a more selective, risk-stratified approach. Surgery is increasingly reserved for cases with documented growth, mass effect, or functional deficits.

**Supplementary Information:**

The online version contains supplementary material available at https://doi.org/10.1007/s11060-026-05713-1.

## Introduction

Intracranial meningiomas are the most common primary intracranial tumors [1]. Due to their typically benign, indolent nature, detection rates rise with age and have increased globally since 2000 [2–6]. In Finland, the detection rate rose from 1953 until the early 2000s and has since remained relatively stable [4, 7]. True prevalence likely exceeds these figures; incidental detection is increasingly common, reaching 1.6–1.8% in older populations [8, 9].

Finnish life-expectancy increased from 75.5 years for men and 82.3 for women in 2005 to 79 and 84.2, respectively, in 2023 [10]. Simultaneously, head CT and brain MRI utilization rose by 77% and 173%, respectively [11, 12]. This combination of population aging and neuroimaging availability suggests a growing volume of incidental meningiomas.

Given the rising prevalence of meningiomas, an increase in surgical interventions might be anticipated, yet reported trends in surgical management are inconsistent. An U.S. study (2004–2012) reported increasing conservative management, with observation rates increasing and surgical resection incidence declining proportionally, although the absolute number of surgeries remained stable [5]. Interestingly, the detection rate of small meningiomas (< 2 cm) rose markedly during this period, possibly due to increased imaging [5]. In contrast, a larger U.S. study (2004–2015) found a declining proportion undergoing surgery (51.1% to 35.6%) but a concurrent increase of nearly 1,500 surgeries annually [13]. However, these data were not age- or sex-adjusted [13].

We examined primary meningioma surgical trends at a Finnish tertiary academic hospital. National registries, which rely heavily on pathologically confirmed cases, likely underestimate the true detection rate [4]. We hypothesized that the age-adjusted surgical intervention rate would not increase between 2005 and 2023, assuming most newly identified meningiomas are small, asymptomatic, and managed conservatively. Conversely, we hypothesized that surgical rates in the elderly would increase as this population becomes healthier and more fit for intervention.

## Methods

### Study design and patient cohort

This retrospective cohort study included all consecutive adults (≥ 18 years) undergoing primary surgical resection for histopathologically confirmed intracranial meningioma at Helsinki University Hospital (HUS) between 2005 and 2023, corresponding to the introduction of the electronic health record system (Uranus) and the most recent complete calendar year at data collection. HUS is the sole provider of intracranial surgery for a catchment area of 2.2 million people.

Data were collected from three electronic health records: Uranus (CGI, Helsinki, Finland), Opera (GE Healthcare, Chicago, IL, USA), and Apotti (Oy Apotti Ab, Helsinki, Finland). Patients were identified by ICD-10 diagnosis code D32 [14], and the meningioma diagnosis histopathologically confirmed. Patient data included general visit notes from consultations conducted by neurosurgeons or neurologists, and data was collected as a retrospective review of clinic visit notes. Radiological data were extracted from local picture archiving and communication system (PACS) [15].

Data were processed within a secure, cloud-based institutional environment (*HUS Acamedic*, https://www.hus.fi/en/research-and-education/hus-acamedic-secure-operating-environment#secure-operating-environment) and pseudo-identifiers were used to ensure patient anonymity.

The study was approved by the institutional review board of Helsinki University Hospital (HUS/216/2023). As a retrospective registry study using pseudonymised data, individual informed consent was waived in accordance with Finnish legislation on the secondary use of health and social data (Act 552/2019).

### Inclusion criteria

Patients required primary surgical treatment for a histopathologically confirmed meningioma at age ≥ 18 years. Patients with prior surgery for the same tumor were excluded. Multiple meningiomas resected in a single procedure were counted as one surgery; meningiomas at different locations treated at different times were counted as separate primary surgeries.

### Surgical indication

Indications were categorized into six non–mutually exclusive groups: symptomatic (neurological deficits/seizures); prophylactic (no significant symptoms, decision based on perceived growth risk, often young patients with small meningiomas); documented growth during follow-up; large tumor size (substantial volume and mass effect, assessed clinically and radiographically without absolute size criteria); suspected malignancy or diagnostic uncertainty (radiologic features suggesting aggressive behavior or requiring histological confirmation); and patient preference (surgery based solely on the patient’s request, most often anxiety, absent any concurrent clinical or radiological indication in the preoperative notes). Classification of each patient was determined through a review of preoperative clinical documentation, including neurosurgical consultation notes and imaging reports. Indications reflected the primary rationale documented by the operating surgeon as the justification communicated to the patient. As all clinical documentation in Finland is accessible to patients through the national Kanta archive, these records represent the surgeon’s explicit, patient-facing reasoning rather than retrospective reinterpretation by the authors.

### Rate of surgery

Population data (sex, age, calendar year) for the HUS catchment area were obtained from the Sotkanet Indicator Bank (https://sotkanet.fi/sotkanet/fi/index), an information service maintained by the Finnish Institute for Health and Welfare [16]. To calculate the incidences, we used the Helsinki University Hospital’s catchment area (HUS ERVA) as reference from the Sotkanet Indicator Bank (16). Surgical incidences were analyzed by 10-year age groups and sex and further stratified by surgical indication. Given the predefined age stratifications (5- or 10-year groups) available in the Sotkanet Indicator Bank, the lower age limit for all analyses using population data was 20 years.

### Statistical analysis

Descriptive statistics, including patient counts and percentages, are presented in tables. Where applicable, interquartile ranges (IQRs) are reported to illustrate the distribution of continuous variables.

Trends in surgical activity and patient characteristics were analyzed using univariate linear regression, with results presented as the unstandardized regression coefficient (*B*), 95% confidence interval (95% CI) and p-values. A p-value < 0.05 was considered statistically significant. Volumetric data were available for 1,030 of 2,278 patients, as imaging was accessible only for studies performed in HUS, not for those obtained externally from other hospitals of the catchment area.

**Fig. 1:**
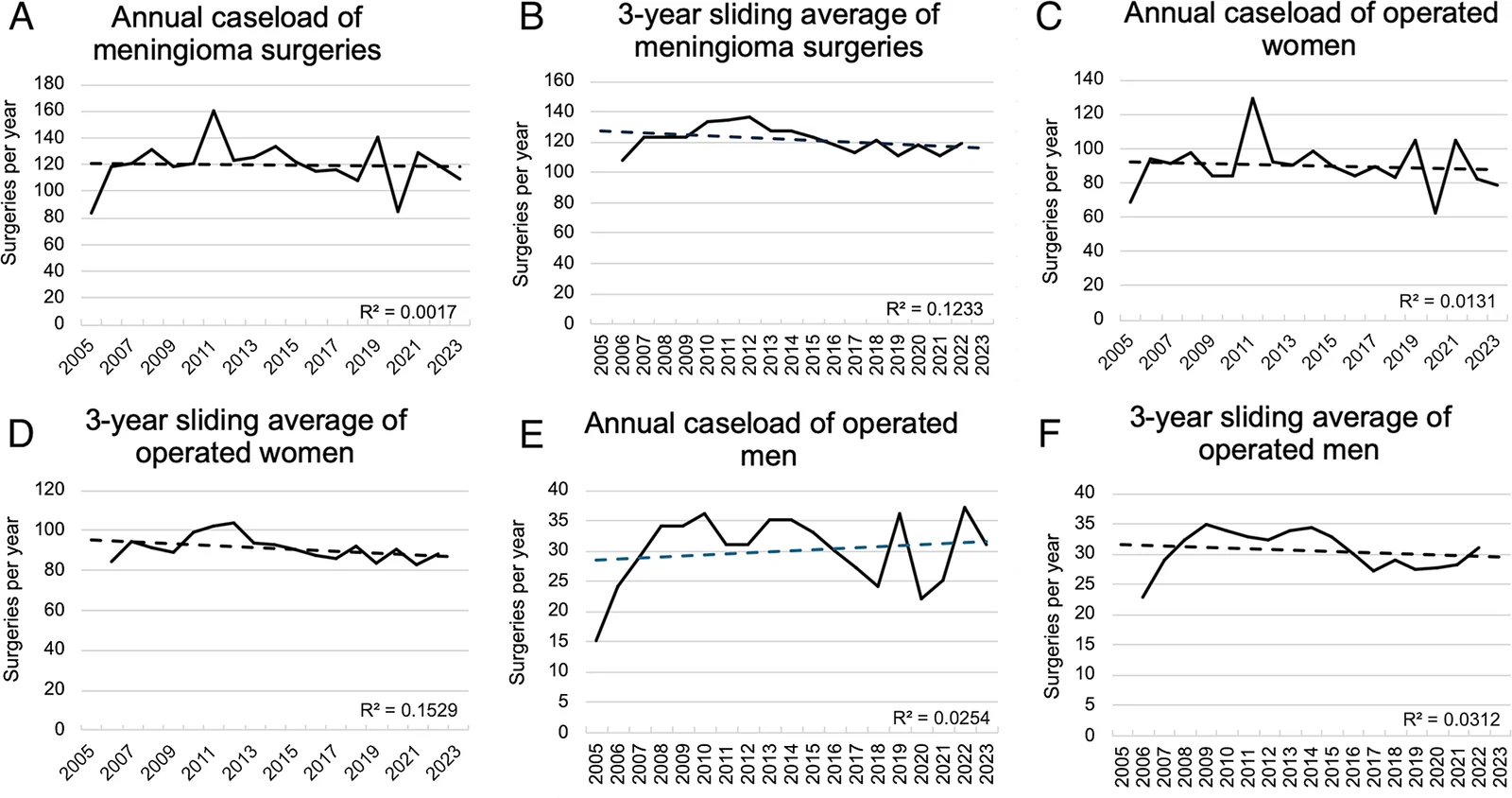
Annual caseloads of meningioma surgeries. (**A**) Overall annual caseload (*p* = 0.868); (**B**) overall 3-year sliding average (*p* = 0.167); (**C**) annual caseload, women (*p* = 0.64); (**D**) 3-year sliding average, women (*p* = 0.12); (**E**) annual caseload, men (*p* = 0.515); (**F**) 3-year sliding average, men (*p* = 0.501). R² denotes the coefficient of determination from linear regression, indicating the proportion of variance in caseload explained by calendar year. Regression coefficients and confidence intervals are provided in Supplementary Table 2

**Fig. 2:**
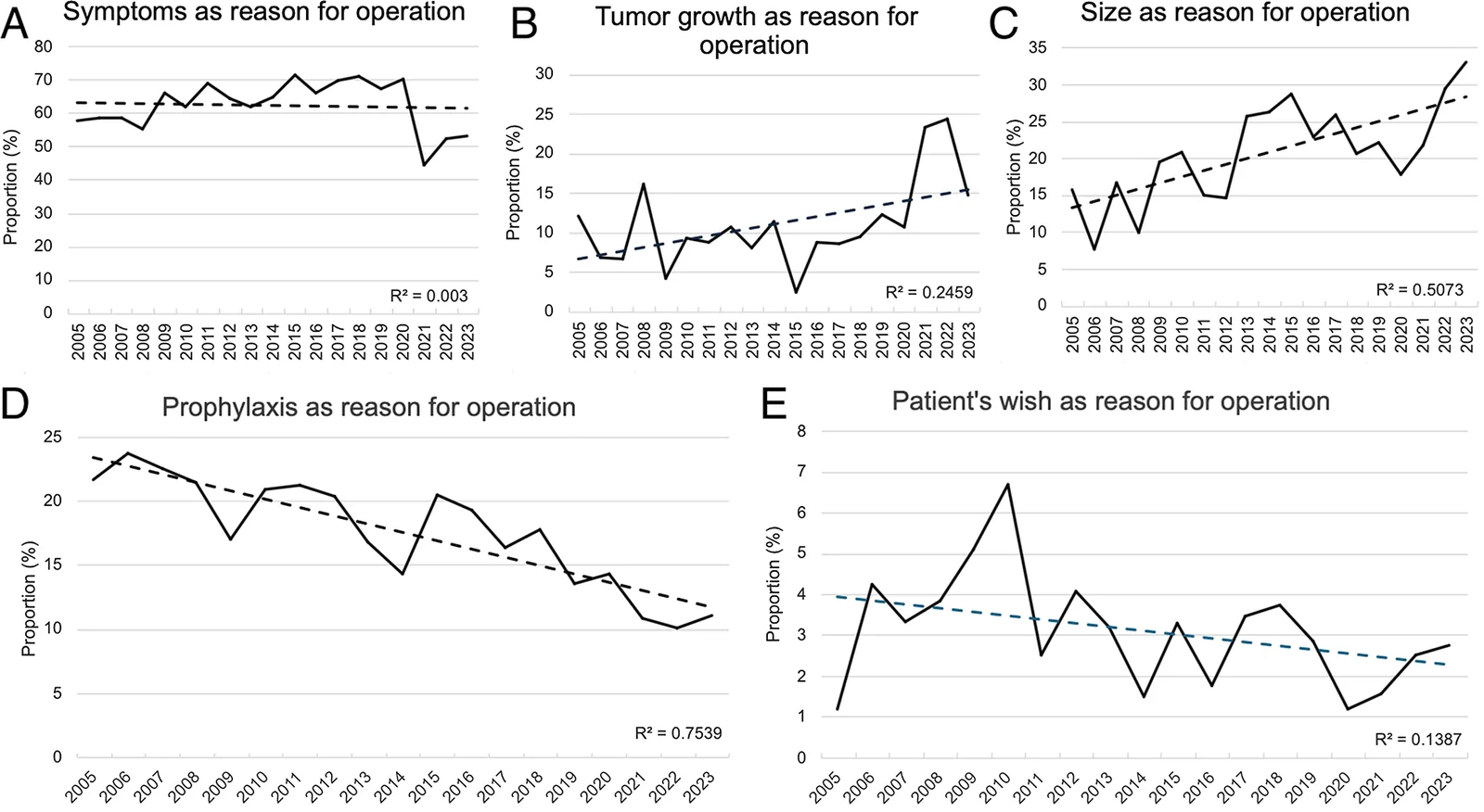
Surgical indications as annual percentages. (**A**) Symptoms (*p* = 0.868); (**B**) tumour growth (*p* = 0.031); (**C**) size (*p* < 0.001); (**D**) prophylaxis (*p* < 0.001); (**E**) patient’s wish (*p* = 0.11). Regression coefficients and confidence intervals are provided in Supplementary Table 2

**Fig. 3:**
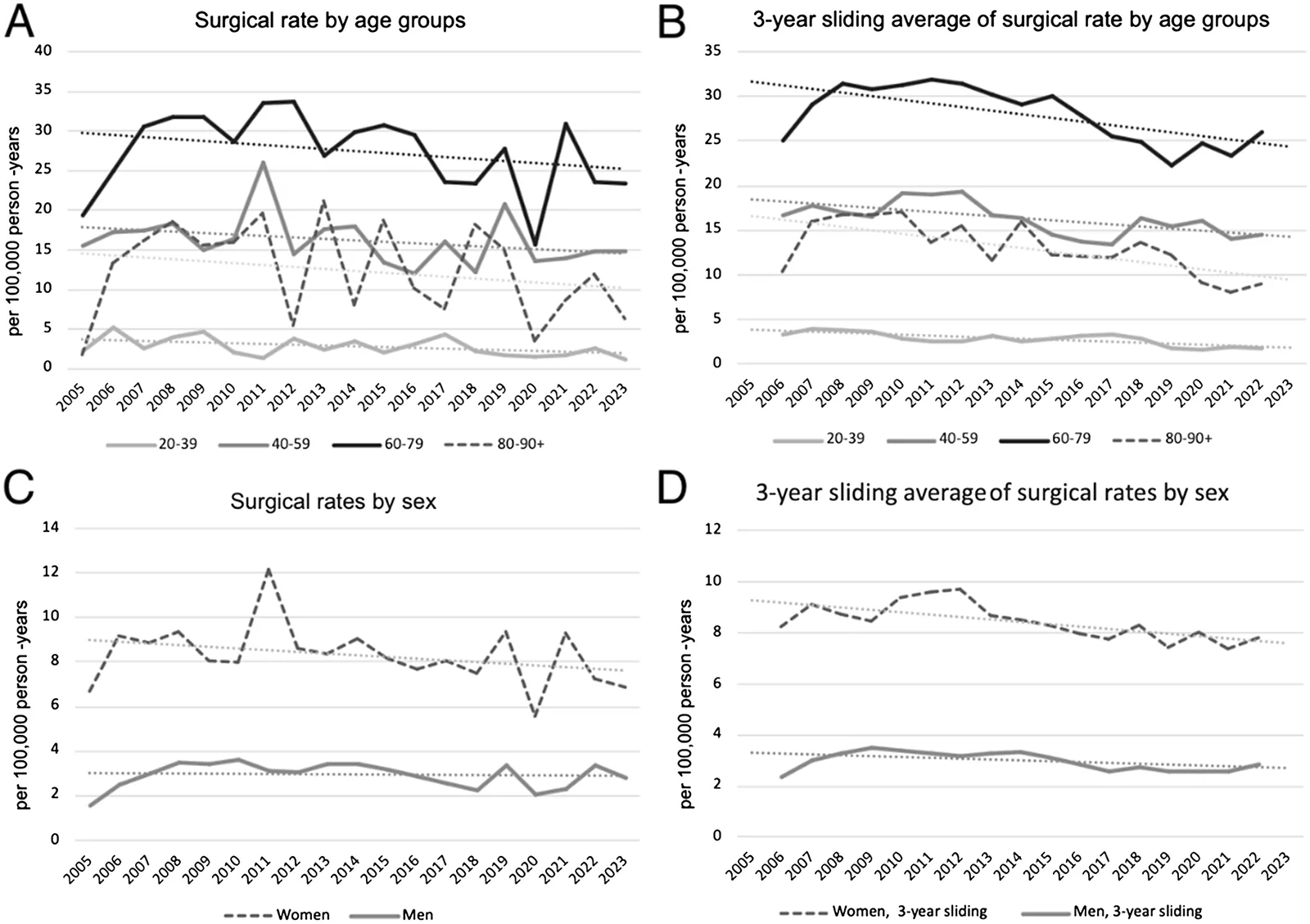
Catchment area age-categorised and sex-stratified incidences of meningioma surgeries. (**A**) Annual by age group (20–39: *p* = 0.033; 40–59: *p* = 0.178; 60–79: *p* = 0.223; 80–90+: *p* = 0.351); (**B**) 3-year sliding averages by age group (20–39: *p* < 0.001; 40–59: *p* = 0.006; 60–79: *p* = 0.005; 80–90+: *p* = 0.003); (**C**) annual by sex (women: *p* = 0.184; men: *p* = 0.82); (**D**) 3-year sliding averages by sex (women: *p* = 0.002; men: *p* = 0.059). Regression coefficients and confidence intervals are provided in Supplementary Table 2

**Fig. 4:**
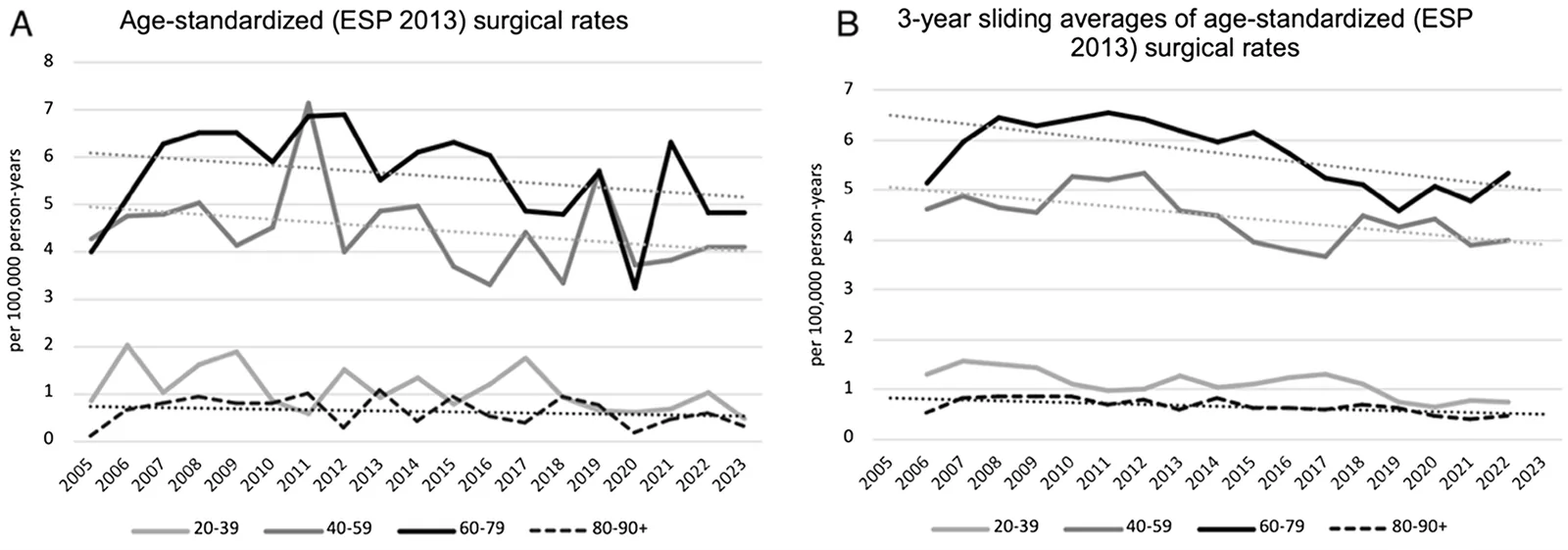
Age-standardised (ESP 2013) surgical incidences by age group. (**A**) Annual values (20–39: *p* = 0.036; 40–59: *p* = 0.182; 60–79: *p* = 0.221; 80–90+: *p* = 0.346); (**B**) 3-year sliding averages (20–39: *p* < 0.001; 40–59: *p* = 0.006; 60–79: *p* = 0.005; 80–90+: *p* = 0.003). ESP = European Standard Population. The ESP-standardised analysis was adjusted for age only; sex-specific standardisation was not performed. Regression coefficients and confidence intervals are provided in Supplementary Table 2

**Fig. 5:**
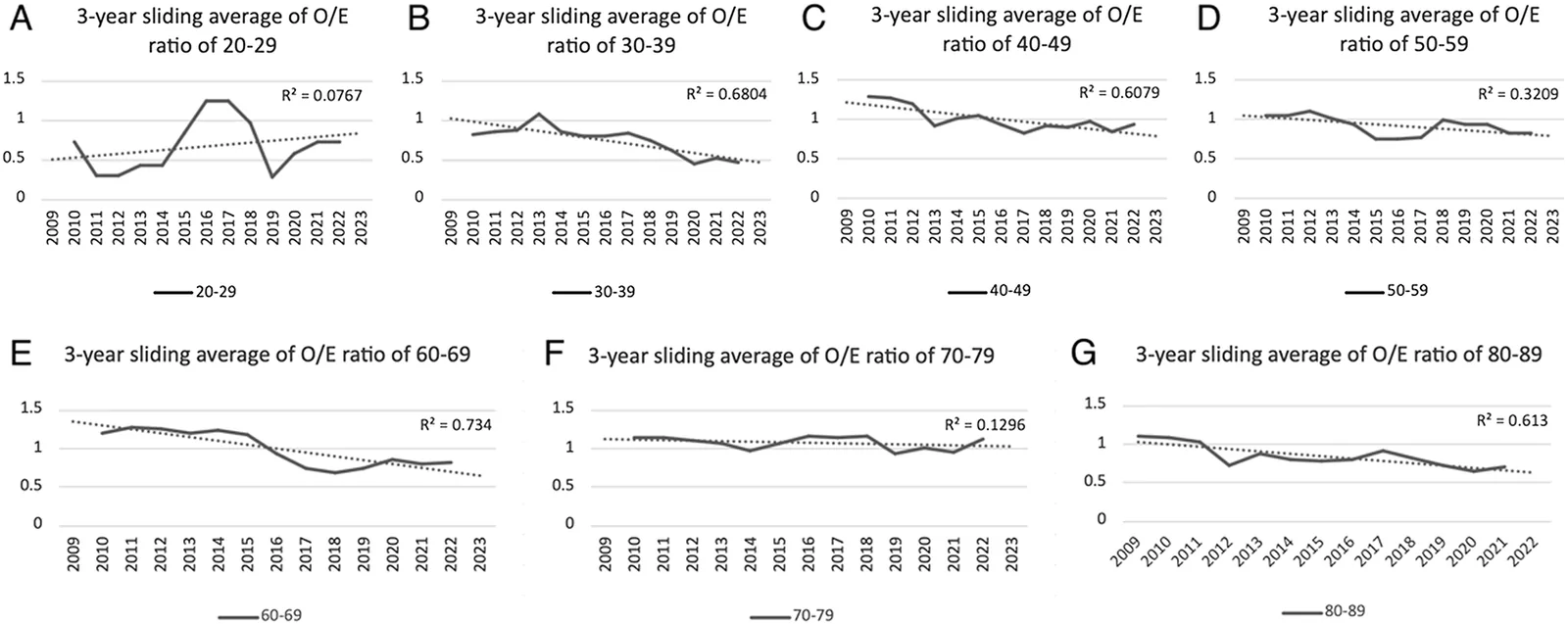
3-year sliding averages of the observed-to-expected ratio of meningioma surgeries by age group. (**A**) 20–29 (*p* = 0.36); (**B**) 30–39 (*p* < 0.001); (**C**) 40–49 (*p* = 0.002); (**D**) 50–59 (*p* = 0.044); (**E**) 60–69 (*p* < 0.001); (**F**) 70–79 (*p* = 0.227); (**G**) 80–89 (*p* = 0.002). Regression coefficients and confidence intervals are provided in Supplementary Table 2

Trends were assessed using both crude annual values (unsmoothed, independent yearly observations) and, for population-based incidence and rate outcomes (caseloads, catchment-area and ESP-standardised incidences, and O/E ratios), 3-year sliding averages to mitigate the year-to-year variation inherent to single-institution counts; both are reported side by side (Figs. 1, 3, 4 and 5), with the sliding averages taken as the primary trend measure. Sliding averages were not applied to proportional indication data (Fig. 2) or patient-level volumetric analyses, which were assessed annually. Reporting both demonstrates the consistency of directional trends across methods rather than selecting a single favourable analysis.

For surgical rate calculations, patients were stratified by sex, WHO tumor grade, and age group. We calculated the incidences of meningioma surgery per 100,000 person-years by age deciles (20–29, 30–39, 40–49, 50–59. 60–69, 70–79, 80–89, 90+). These age groups are based upon the European Standard population (ESP) [17]. We used the ESP-weighted direct age standardization to derive age-standardized incidence estimates by calendar year. Age standardization using the ESP 2013 was applied to account for the progressive aging of the catchment area population over the study period. Since meningioma incidence rises steeply with age, a growing older population would produce more surgeries over time even without changes in management; the ESP serves as a fixed age-weight reference to remove this confound. The ESP is used purely as a methodological tool and does not imply generalizability beyond our catchment area.

The expected number of surgeries per age group and period (2009–2011 through 2021–2023) was calculated by applying the age-specific 2005–2008 baseline rate to each period’s population; the observed-to-expected (O/E) ratio divided observed by expected surgeries. The 90 + group was excluded (zero baseline incidence). The 2005–2008 baseline represents the earliest complete time period following the new electronic health record system (Uranus) introduction, predating widespread active surveillance; this may reflect less selective early practice, potentially amplifying the apparent magnitude of subsequent decline.

We adhered to the STrengthening the Reporting of OBservational (STROBE) studies in Epidemiology guidelines.

## Results

### Patient cohort

The cohort comprised 2,278 patients (75% women; 3:1 ratio) with a median age of 61 years (IQR 51–71), females being slightly younger than males (median 60 vs. 63 years).The median age of operated patients increased significantly over the study period, from 57 years in 2005 to 63 years in 2023, corresponding to a gain of 2.9 months per calendar year (Supplementary Fig. 1). Absolute annual surgical caseload remained stable (range: 83–160 cases) with no significant sex-specific trends (Fig. 1). Among patients aged ≥ 70 years, the absolute annual number of surgeries increased significantly over the study period (0.97 surgeries/year, *p* = 0.006), whereas no significant change was observed in patients aged ≥ 80 years (*p* = 0.788); age within the ≥ 70 years subgroup did not increase (median 76 years, stable across the period; mean-age regression in Supplementary Table 1).

### Surgical indications

Symptoms were the primary indication (62%), most commonly seizures (19%), visual deficits (14%), and cognitive impairment (12%); other indications included large tumor size (21%) and documented growth (11%), with 30% asymptomatic at surgery (Supplementary Table 3).

Over the study period, the annual proportion presenting with visual symptoms increased significantly while other symptoms remained stable (Supplementary Fig. 2). Surgeries for tumor growth and large size increased significantly, whereas prophylactic surgeries declined (Fig. 2).

### Catchment area surgical incidences

Crude annual surgical incidence declined significantly only in patients aged 20–39. Because single-institution counts are subject to substantial year-to-year variation, trends were assessed primarily from 3-year sliding averages; consistent with the annual direction, these showed significant declines in all age groups, in women, and a near-significant decline in men (Fig. 3).

### ESP-standardized surgical incidences

Age-standardized (ESP 2013) annual incidences were stable in all age groups except 20–39 years (significant decline), whereas the 3-year sliding averages showed decreasing trends in the same direction across all age groups (Fig. 4).

### Observed versus expected surgical incidences

A separate demographic-adjusted analysis compared observed surgical incidences with expected rates derived from the 2005–2008 baseline, accounting for changes in age and sex. Annual O/E ratios declined significantly in the 30–39 and 60–69 groups (Supplementary Fig. 3); the 3-year sliding-average analysis, prioritised for trend assessment as above, showed significant decreases in the same direction across all groups except 20–29 and 70–79 (Fig. 5).

### Tumour volume sub-analysis

Patients with volumetric data available were younger and clustered in earlier study years, and more often had size-related, prophylactic, or patient-preference indications (all *p* < 0.05), but did not differ in sex or WHO grade (Supplementary Table 4). Median volume was larger for supratentorial than infratentorial tumours (10.5 vs. 9.0 cm³), and among supratentorial lesions, larger for non-skull base than skull base tumours (13.3 vs. 8.6 cm³). In annual log-linear regression, overall tumour volume increased significantly by an estimated 2.5% per year (95% CI 0.5–4.7, *p* = 0.015), a trend driven by supratentorial tumours (*p* = 0.047). In contrast, among patients operated on for large tumour size, volume did not change significantly over time (− 2.0% per year, *p* = 0.191).

## Discussion

This retrospective study of almost 2300 patients reveals multiple temporal shifts in surgically treated intracranial meningiomas. While absolute annual caseloads remained stable, age-adjusted and ESP-standardized analyses demonstrate a true reduction in surgical intervention relative to the aging population. The sex-stratified reduction was significant in women and nearly significant in men (*p* = 0.059), suggesting a trend toward declining incidence in both sexes, though the evidence is stronger for women.

The decline in O/E ratios aligns with age-adjusted and ESP-standardized trends, confirming reduced surgical utilization. As contemporary management spans multiple modalities, these data cannot establish whether overall treatment activity has changed. In patients aged 30–69 years, this likely reflects the global shift toward watchful waiting or active surveillance for small, incidental meningiomas, thereby reducing the proportion of patients selected for surgery. In patients aged 80–89 years, the decline is likely multifactorial, driven by a stricter perioperative risk stratification and an increasing use of non-surgical modalities, such as targeted radiotherapy, which are not captured in surgical registries.

Two age groups (20–29 and 70–79 years) maintained stable O/E ratios. In the 20–29 group, this likely reflects a small subset presenting with unequivocal symptoms and clear indications, where the necessity of intervention is consistently high. In the 70–79 group, this likely reflects a large age band in which patients remain suitable surgical candidates, sustaining procedure numbers. The relative preservation of surgical activity in older meningioma patients is consistent with findings reported previously [18]. However, as both groups showed declining ESP-standardized incidences, the stable O/E ratios likely reflect similar 2005–2008 baseline activity rather than truly sustained rates. These age-specific interpretations should be treated with caution given small numbers and wide confidence intervals, and remain hypothesis-generating.

Our findings are partially explained by evolving surgical indications: surgeries for tumor growth and large size increased significantly, while prophylactic procedures and ‘patient wish’ declined. Consistent with this, operated tumour volume increased significantly over time (an estimated 2.5% per year), supporting a shift toward more selective surgical management in which resection is increasingly reserved for larger lesions. Notably, tumour volume within the “large size” indication category itself remained stable, suggesting that while surgical selection became more restrictive overall, the volume threshold prompting size-driven resection did not change.

Concurrently, the median age of the surgical cohort increased significantly, driven by the general aging of the Finnish population and enabled by advances in neuroanesthesia and perioperative care that broadened surgical eligibility in older patients [19–22]. Our hypothesis that surgical activity in the elderly would increase was only partially supported. While the absolute number of surgeries in patients aged ≥ 70 years rose significantly, population-adjusted incidence nonetheless declined or remained stable, indicating that the growth in elderly surgical volume did not keep pace with the expansion of the elderly population itself. The absence of a comparable increase in patients aged ≥ 80 years suggests that the very elderly remain a carefully selected surgical group. Together with the concurrent rise in SRS and active surveillance, these findings indicate that improved surgical fitness in older patients has been offset by more selective, population-level management, consistent with contemporary guidelines recommending observation for asymptomatic tumors [23, 24], and despite increased neuroimaging and incidental detection [5, 11–13].

Visual symptoms as a presenting complaint increased significantly, suggesting that meningiomas in vision-critical locations, such as the optic nerve sheath, tuberculum sellae [25], or medial sphenoid wing, are less amenable to conservative management given the risk of irreversible loss of vision. Improved access to neuro-ophthalmological assessment may also contribute to this increase. Conversely, stable rates for major neurological deficits and for seizure-indicated surgeries, seizures being a well-recognized presenting symptom and established surgical indication [26, 27], suggest consistent intervention thresholds throughout the study period.

In contrast to previous studies [5, 13, 28], we accounted for age and population demographics, which changes conclusions: crude incidences appeared stable, but after adjusting for the changing size of age groups, standardized incidences in both younger and elderly cohorts were consistently lower than nearly 20 years earlier. To our knowledge, no comparable age- and demographically-adjusted studies of meningioma surgical incidence exist.

Two demographically adjusted cohort studies yielded conflicting results. A U.S. analysis of over 44,000 microscopically confirmed meningiomas found no significant change in incidence during 2004–2009 [29], whereas an Australian study (2000–2008, > 1,800 patients) reported an increase, specifically among male patients [30]. Neither adjusted for patient age, a crucial consideration for long-term trend analysis. The selective-management trend in our cohort is consistent with U.S. studies reporting a declining proportion of patients undergoing surgery alongside increasing observation [5, 13].

Among unadjusted studies, a Swedish cohort (1999–2017) reported increased surgical incidence in patients over 65 years [28], likely reflecting demographic aging. Two large U.S. studies reported declining proportions undergoing surgery, from 48.8% to 38.3% alongside rising observation [5], and a decreasing proportional rate despite an increasing absolute number of operations, attributed to greater incidental detection [13]. Again, none adjusted for age or demographics, a key methodological difference from our analysis. Emerging molecular and methylation-based classification systems may refine surgical decision-making by identifying tumours at higher recurrence risk independent of WHO grade [31]. Though not in routine use during our study, future incidence studies will need to account for their influence on operative thresholds.

### Study strengths and limitations

One of the strengths of this study is that it minimizes the selection and referral biases as a single-center study design of an academic hospital serving a population-based cohort together with a universal healthcare system. We were able to analyze all patient records manually and data were collected in a structured and systematic way based on predefined criteria. Moreover, a large study population and the long-term study period of 19 years provides sufficient duration for detection of temporal trends. Another major strength is, that due to comprehensive Finnish data registries we were able to take into account populational changes affecting the incidence of meningiomas.

This study also has several limitations. As a retrospective study, it is susceptible to documentation and classification bias, particularly in assigning surgical indications across 19 years of evolving documentation practices; formal inter-rater reliability was not assessed. The lack of a comparative non-surgical cohort limits our ability to quantify the overall management shift. Our unit captures all surgically treated meningiomas, but not all diagnosed meningiomas — particularly small or incidental lesions in elderly or comorbid patients — are referred to us; consequently, the number of diagnosed but non-operated meningiomas, and thus the true institutional proportion undergoing surgery, cannot be established. However, nearly all younger (< 70 years) and otherwise healthy patients with incidental meningiomas are referred for a surveillance decision, so trends in younger age groups are likely accurate, whereas ascertainment in the oldest and most comorbid patients is less complete and warrants greater caution. Some patients may also have sought radiological diagnosis outside HUS, introducing modest referral bias. A decline in overall surgical incidence rather than absolute case volume could potentially reflect a general reduction in neurosurgical activity. However, this explanation does not apply to our department. The observed reduction reflects population-adjusted meningioma surgical incidence specifically and is unlikely to stem from a general decline in overall neurosurgical activity at our clinic. Although surgical volume fell temporarily during the COVID-19 pandemic (2020–2021), no persistent trend change is apparent on inspection of Fig. 1; a formal sensitivity analysis excluding these years was not performed. Surgical decisions at our institution reflect the independent judgement of individual surgeons (more than 40 over the study period), based on symptoms, patient preference, and imaging, rather than a protocolled guideline. As meningioma management is not routinely governed by neuro-oncological multidisciplinary review at our institution, observed changes cannot be attributed to discrete policy shifts, but likely reflect gradual, surgeon-level assimilation of evolving evidence toward more selective management. SRS has become an increasingly used modality for small-to-medium meningiomas, particularly in eloquent locations or higher-risk patients [23, 32], with growing evidence, including the IMPASSE study, supporting it as effective primary treatment [33]. Finally, some patients previously referred for resection may now be managed with SRS, contributing to the observed decline. However, institutional SRS volumes were unavailable, so these data cannot directly quantify changes in overall treatment paradigms. Similarly, temporal trends in tumour location, size, WHO grade, Simpson grade, and perioperative outcomes were not analyzed, limiting interpretation of whether the shift reflects changes in the complexity and risk profile of operated tumours. Both warrant dedicated future analyses from our institution.

### Conclusion and future outlook

Over 19 years, absolute surgical caseload remained stable, whereas age-adjusted and population-standardized incidences declined significantly despite the aging cohort and increasing incidental detection. This supports a shift toward a more selective, risk-stratified approach. In the future, we will need evidence-based long-term follow-up protocols for the expanding non-operatively managed meningioma population and surgical competence for elderly comorbid patients with symptomatic or progressive meningiomas.

## Supplementary Information

Below is the link to the electronic supplementary material.


Supplementary Material 1


## Data Availability

The data that support the findings of this study are held within the secure research environment of Helsinki University Hospital (HUS Acamedic). The data are not publicly available due to privacy and ethical restrictions but may be available from the corresponding author on reasonable request and subject to institutional approval.
