# Supplementary material for: Declining age-adjusted surgical incidence of intracranial meningiomas: a 19-year retrospective analysis from an academic hospital
Source: J Neurooncol. 2026 Jul 21;179(1):6. doi: 10.1007/s11060-026-05713-1 (PMC13388540; doi:10.1007/s11060-026-05713-1)
Supplement: Supplementary file 1 — Supplementary Material 1 [file 11060_2026_5713_MOESM1_ESM.pdf]

SUPPLEMENTARY FILES

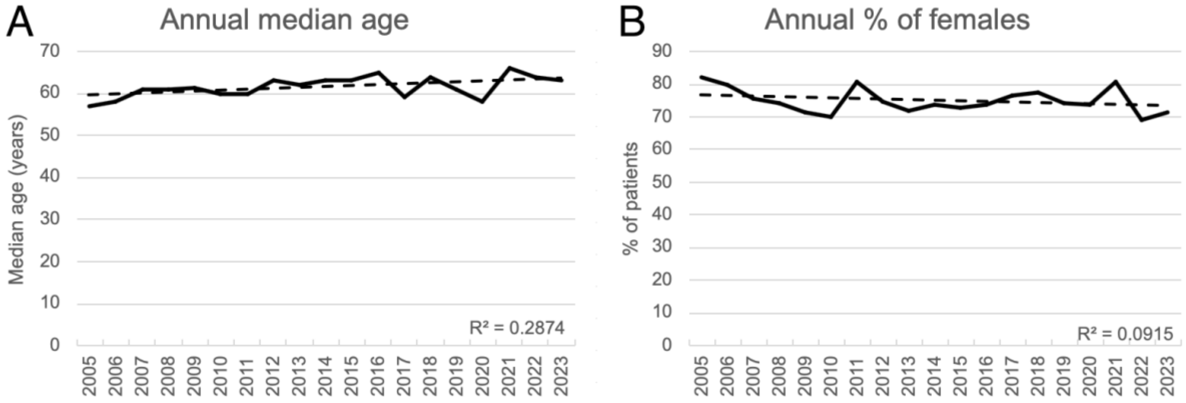

Supplementary Fig 1 A) The annual median age of operated meningioma patients (*B* = 0.236, 95% CI (0.046 to 0.426), p=0.018). B) The annual percentage of operated women (*B* = -0.201, 95% CI (-0.525 to 0.123), p=0.208).

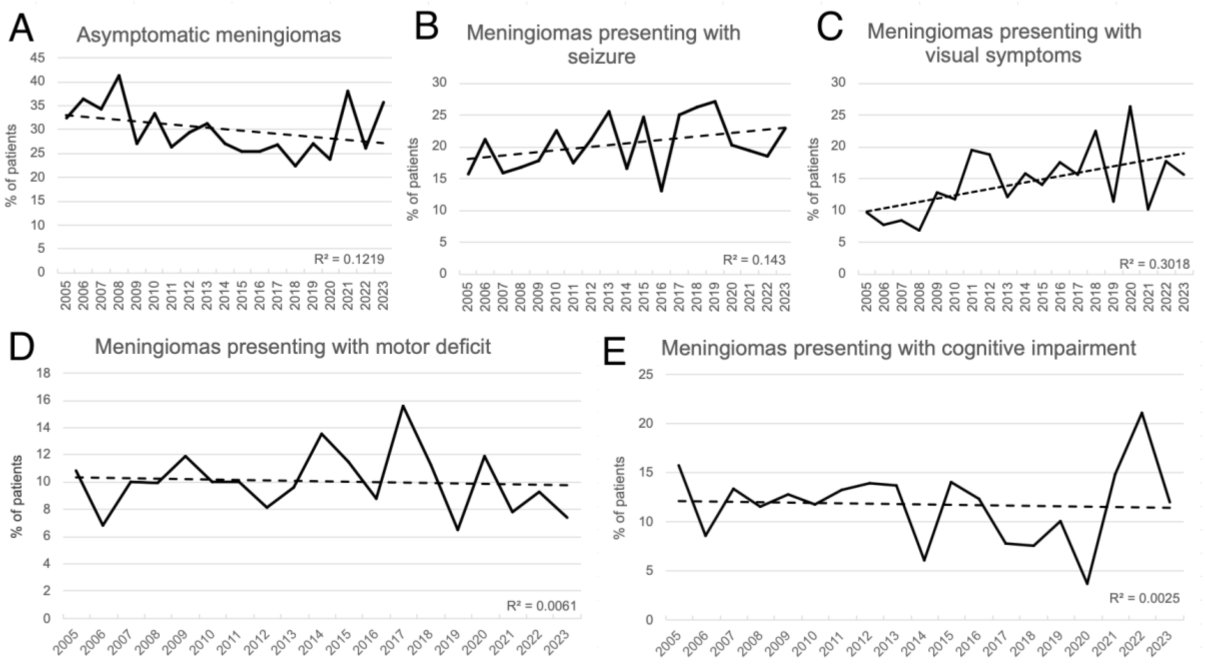

Supplementary Fig 2 The annual percentage of operated patients with A) no symptoms (*B* = -0.328, 95% CI (-0.779 to 0.123), p=0.143), B) seizures (*B* = 0.274, 95% CI (-0.07 to 0.618), p=0.111), C) visual symptoms (*B* = 0.503, 95% CI (-0.111 to 0.894), p=0.015), D) motor deficits (*B* = -0.032, 95% CI (-0.241 to 0.177, p=0.75) and E) cognitive impairment (memory or personality changes) (*B* = -0.035, 95% CI (-0.390 to 0.320), p=0.837)

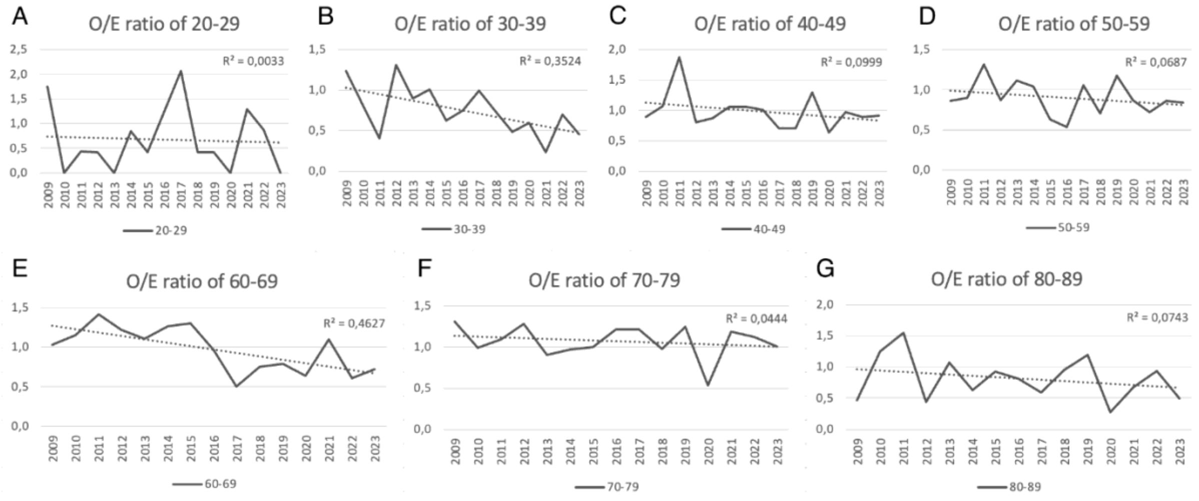

Supplementary Fig 3 Annual observed-to-expected ratio of meningioma surgeries in the age groups of A) 20-29 (B=-0.005, 95% CI (-0.093 to 0.082), p=0.897) B) 30-39 (B=-0.038, 95% CI (-0.071 to -0.005), p=0.026) C) 40-49 (B=-0.023, 95% CI (-0.062 to 0.016), p=0.223) D) 50-59 (B=-0.012, 95% CI (-0.041 to 0.016), p=0.362) E) 60-69 (B=-0.042, 95% CI (-0.071 to -0.013), p=0.008) F) 70-79 (B=-0.01, 95%_CI (-0.037 to 0.017), p=0.435) G) 80-89 (B=-0.023, 95% CI (-0.07 to 0.024), p=0.305). The expected number of surgeries per age group was calculated by applying the age-specific 2005–2008 baseline rate.

Supplementary Table 1. Trends in surgical activity among elderly patients (2005–2023)

Analysis	n	B (change per calendar year)	95% CI	R^2	p-value
Absolute number of surgeries, ≥70 years	629	+0.97 surgeries	+0.31 to +1.62	0.365	0.006
Absolute number of surgeries, ≥80 years	158	+0.04 surgeries	−0.28 to +0.37	0.004	0.788
Mean age within ≥70 years subgroup	629	−0.07 years	−0.14 to −0.001	0.006	0.046

B represents the estimated annual change derived from linear regression against calendar year. Absolute surgery counts were modelled per calendar year (n = 19 years). Mean age was modelled at the patient level within the ≥70 years subgroup; the median age within this subgroup was similarly stable (76 years overall, range 73–78 across the study period).

Supplementary Table 2. Regression statistics for Figures 1–5

Figure	Panel / Group	B	95% CI
Fig 1	A) Overall annual caseload	−0.126	−1.703 to 1.45
	B) Overall 3-yr sliding average	−0.587	−1.449 to 0.274
	C) Women, annual	−0.291	−1.583 to 1.00
	D) Women, 3-yr sliding average	−0.474	−1.086 to 0.138
	E) Men, annual	0.165	−0.358 to 0.688
	F) Men, 3-yr sliding average	−0.114	−0.464 to 0.237
Fig 2	A) Symptoms	−0.072	−0.748 to 0.604
	B) Tumour growth	0.49	0.051 to 0.929
	C) Size	0.838	0.415 to 1.26
	D) Prophylaxis	−0.652	−0.842 to −0.461
	E) Patient's wish	0.139	−0.210 to 0.250
Fig 3	A) Annual, 20–39	−0.104	−0.199 to −0.009
	A) Annual, 40–59	−0.188	−0.469 to 0.094
	A) Annual, 60–79	−0.254	−0.679 to 0.17
	A) Annual, 80–90+	−0.237	−0.758 to 0.284
	B) 3-yr sliding, 20–39	−0.11	−0.159 to −0.061
	B) 3-yr sliding, 40–59	−0.232	−0.387 to −0.077
	B) 3-yr sliding, 60–79	−0.409	−0.672 to −0.145
	B) 3-yr sliding, 80–90+	−0.393	−0.628 to −0.158
	C) Annual, women	−0.078	−0.196 to 0.041
	C) Annual, men	−0.006	−0.057 to 0.046
	D) 3-yr sliding, women	−0.096	−0.152 to −0.039
	D) 3-yr sliding, men	−0.033	−0.068 to 0.002
Fig 4	A) Annual, 20–39	−0.040	−0.078 to −0.003
	A) Annual, 40–59	−0.051	−0.129 to 0.026
	A) Annual, 60–79	−0.052	−0.139 to 0.034
	A) Annual, 80–90+	−0.012	−0.038 to 0.014
	B) 3-yr sliding, 20–39	−0.043	−0.062 to −0.024
	B) 3-yr sliding, 40–59	−0.064	−0.106 to −0.021
	B) 3-yr sliding, 60–79	−0.084	−0.139 to 0.03
	B) 3-yr sliding, 80–90+	−0.02	−0.031 to −0.008
Fig 5	A) 20–29	−0.024	−0.031 to 0.079
	B) 30–39	−0.039	−0.057 to −0.021
	C) 40–49	−0.031	−0.047 to −0.014
	D) 50–59	−0.018	−0.035 to −0.001
	E) 60–69	−0.051	−0.071 to −0.03
	F) 70–79	−0.008	−0.021 to 0.005
	G) 80–89	−0.03	−0.046 to −0.014

B = unstandardised regression coefficient; CI = confidence interval. Corresponding p-values are shown in the respective figure legends.

Supplementary table 3: Presenting symptoms and surgical indications.

Symptoms	1595 (70)
Asymptomatic (%)	683 (30)
Seizure (%)	443 (19)
Visual symptoms (%)	325 (14)
Cognitive impairment (memory or personality changes) (%)	271 (12)
Motor deficit (%)	228 (10)
Other symptoms (%)	328 (14)
**Surgical indications**	
Symptoms (%)	1418 (62)
Size (%)	473 (21)
Growth in follow-up (%)	250 (11)
Prophylactic (%)	399 (18)
Patients wish (%)	71 (3)
Diagnosis/suspected malignancy (%)	59 (3)
Same time with another tumor/aneurysm (%)	18 (1)

Data presented as number of patients (%). N=2278

Supplementary Table 4. Tumour volume by location and temporal trends (2005-2023)

	n (total)	Volumetric data, n (%)	Median volume, cm^3 (IQR)	Min–Max, cm^3	Annual change (%)	95% CI (%)	R^2	p-value
By location								
Supratentorial	1,933	895 (46.3%)	10.5 (28.7)	0.1–532.7	+2.2	0.0 to +4.5	0.004	0.047
Non-skull base	971	509 (52.4%)	13.3 (33.1)	0.2–177.1	+2.8	−0.1 to +5.9	0.007	0.060
Skull base	823	348 (42.3%)	8.6 (24.4)	0.1–138.6	+2.0	−1.4 to +5.6	0.004	0.251
Infratentorial	316	125 (39.6%)	9.0 (21.1)	0.3–93.7	+2.7	−2.9 to +8.7	0.007	0.342
Both	29	12 (41.4%)	39.7 (76.4)	2.8–116.7	—*	—	—	—
All patients	2,278	1,030 (45.2%)	—	—	+2.5	+0.5 to +4.7	0.006	0.015
**By surgical indication**								
Large tumor size	—	239	—	—	-2.0	-4.9 to +1.0	0.007	0.191

**The "Both" group (n=12) was excluded from regression due to insufficient sample size. Annual change was derived from patient-level log-linear regression of tumour volume against calendar year; 3-year sliding averages were not applied to volumetric analyses.*
